# Psychometric properties of the Turkish version of the VARK learning style inventory for athletes

**DOI:** 10.1186/s13102-026-01529-8

**Published:** 2026-01-17

**Authors:** Üstün Türker, Mustafa Barış Somoğlu, Mustafa Koç, Özgür Bostancı

**Affiliations:** 1 Department of Physical Education and Sports, Faculty of Sports Sciences, Gümüşhane University, Gümüşhane, Turkey; 2 Department of Physical Education and Sports, Faculty of Sports Sciences, Ondokuz Mayis University, Samsun, Turkey

**Keywords:** Confirmatory factor analysis, Measurement, Psychometrics, Validity and reliability, VARK

## Abstract

**Background:**

Understanding how athletes learn and recognizing their learning preferences are important cognitive and sensory components that may support effective instructional planning in sport settings. This study aimed to adapt the VARK Learning Style Inventory for Athletes (Visual, Aural, Read/Write, and Kinesthetic modalities) into Turkish and to evaluate its psychometric properties.

**Methods:**

A total of 854 licensed athletes from individual and team sports in Türkiye participated in the study. The Turkish version of the VARK Learning Style Inventory for Athletes was administered using a paper-based format. Data were analyzed using IBM SPSS Statistics (v26) and LISREL (v8.80). Construct validity was examined using a Multitrait–Multimethod Confirmatory Factor Analysis (MTMM-CFA) approach with a correlated trait–correlated uniqueness (CTCU) specification, while reliability was assessed using internal consistency and test–retest procedures.

**Results:**

The MTMM-CFA supported the four-factor structure of the inventory, indicating an acceptable model fit and strong associations among the learning style dimensions, consistent with a multimodal learning preference profile. Internal consistency analyses yielded Kuder–Richardson 20 (KR-20) coefficients ranging from 0.574 to 0.623 across subscales, reflecting moderate reliability.

**Conclusions:**

The findings suggest that the Turkish version of the VARK Learning Style Inventory for Athletes demonstrates acceptable psychometric properties, with evidence of construct validity and moderate internal consistency. While the results support its use for assessing learning preferences among Turkish athletes, further research across different sport disciplines and age groups is recommended to strengthen there liability evidence and practical applicability. Overall, the study contributes to measurement practices in sport and exercise sciences by providing a culturally adapted instrument for examining athletes’ learning preferences.

## Introduction

Learning involves relatively stable changes in knowledge, skills, and attitudes that occur through experience and practice. Importantly, the learning process does not operate uniformly across individuals; somewhat, it varies according to differences in cognitive processing, sensory preferences, prior experiences, and motivational characteristics [1, 2]. One of the central constructs used to explain such individual variability is learning style. Learning styles refer to the preferred ways individuals perceive, process, and retain information or skills, often through one or more dominant sensory modalities [1]. From an educational science perspective, learning is widely conceptualized as a multidimensional process shaped by the interaction of cognitive, behavioral, and environmental factors, rather than a uniform or linear mechanism [3]. In applied sport and physical activity settings, these individual differences are particularly salient, as learning frequently occurs under time pressure, physical fatigue, and performance constraints that amplify the role of sensory information processing [4].

Several studies have further demonstrated that learning preferences are shaped by an interaction of cognitive, sensory, and experiential factors across different educational contexts [5–9]. According to Dunn and Griggs, every individual possesses the capacity to learn independently of their academic abilities; however, each person may follow a different path in the learning process, and perceptual preferences can play a critical role in shaping that path [10]. In a study conducted by Türker and Bostancı involving pre-service teachers enrolled in programs of physical education and sports, visual arts, and music, it was reported that although individuals tended to favor a dominant learning style based on their perceptual preferences, they did not exclusively adhere to that single style [9]. Instead, they adopted a multimodal learning model throughout the learning process. Comparable patterns of multimodal learning engagement have been reported in sport pedagogy and coaching research, where athletes are required to integrate visual, auditory, and kinesthetic information simultaneously during skill acquisition [11]. Recent sport psychology research further emphasizes that individual differences in learning and information processing play a central role in athlete development, particularly within coach–athlete instructional interactions and pedagogical decision-making processes [12, 13]. These findings support the notion that learning preferences in sport contexts should be examined within a broader pedagogical and psychological framework that accounts for both individual variability and the instructional environment [14].

At this point, the VARK model, which aims to offer personalized learning experiences, becomes particularly relevant. The VARK model not only categorizes learning preferences into four primary styles (Visual, Aural, Read/Write, Kinesthetic), but also serves as a sensory framework that identifies whether an individual prefers a single dominant style or a combination of two, three, or even all four styles [15]. Despite the widespread use of the VARK framework, existing research has predominantly focused on general educational settings, while comparatively fewer studies have examined its application within athletic contexts. This imbalance is notable given that sport learning environments are fundamentally practice-based and rely heavily on sensory feedback and instructional cues delivered by coaches [16]. Learning in sport settings involves complex interactions between perceptual, cognitive, and motor processes that may differ substantially from traditional classroom-based learning environments [16, 17]. Furthermore, although an athlete-specific version of the VARK inventory has been proposed, its psychometric properties have been evaluated in a limited number of studies, and a validated Turkish adaptation has not yet been reported. Accordingly, there is a need for a careful psychometric examination of the VARK Learning Style Inventory for Athletes within sport-related learning contexts [12, 13].

In sport and physical activity contexts, learning involves integrating cognitive, perceptual, and motor processes under dynamic, time-constrained conditions. From a learning preference perspective, athletes are continuously exposed to visual demonstrations, auditory instructions, written or symbolic feedback, and kinesthetic practice, which directly corresponds to the four sensory modalities proposed in the VARK framework. Consequently, athletes are often required to engage with multiple modalities simultaneously, making multimodal learning profiles particularly relevant in sport education and coaching environments [6, 18]. Empirical studies in motor learning and coaching have demonstrated that the effectiveness of instruction is closely tied to how athletes attend to and integrate these sensory channels during practice [17]. Due to factors such as time pressure, the necessity for rapid decision-making, and reliance on coaching cues, these environments require not only a dominant learning style but also the integration of multimodal learning models that engage multiple sensory systems. Any delay or lack of guidance in communication between coach and athlete may result in performance loss or erroneous motor responses. In this context, identifying athletes' individual learning styles may be a key strategy for personalizing instructional methods, effectively incorporating visual and auditory stimuli, and enhancing both individual and team performance [19]. Such individualized instructional approaches have been highlighted as a core component of effective coaching practice, particularly in high-performance sport settings [11].

At the theoretical core of the VARK model lies the assertion that instructional environments should be organized according to individuals’ sensory preferences to ensure and sustain learning efficiency. This approach supports the design of instructional strategies tailored to diverse learning needs in practice-based disciplines. However, the theoretical and practical applications of learning style models, including VARK, have been the subject of considerable debate and criticism within the scientific literature. Prominent critiques, such as those by Pashler et al. and Cuevas, highlight two primary concerns: the lack of empirical evidence that aligning instruction with individuals’ reported learning styles improves outcomes, and the limited psychometric support for many learning style inventories [20–22]. These critiques have been particularly influential in applied fields such as sport science, where measurement validity is a prerequisite for meaningful interpretation and practical application [23]. In response to these concerns, the present study does not aim to promote the direct effect of the VARK model on performance, but rather to address its methodological shortcomings through rigorous adaptation and validation. Establishing a reliable and valid measurement tool is a prerequisite for meaningful investigations into the model’s potential value in applied contexts such as sports education. Therefore, this study undertakes a comprehensive adaptation and psychometric evaluation of the VARK Learning Style Inventory for Athletes into Turkish, enabling future research to examine how learning preferences interact with other variables in complex, real-world coaching environments.

In the existing literature, most studies using the VARK model have relied on its general version, designed for broad populations rather than specific professional groups [24–32]. Research employing the athlete-specific version of the VARK inventory remains limited [6–8, 15], and to date, no adaptation or validation study of this version has been conducted in Turkey [9, 33]. Lujan and DiCarlo highlighted the importance of cultural and linguistic adaptation in psychometric research, emphasizing that language-related differences may influence reliability coefficients [34]. In light of these considerations, the present study aims to adapt the VARK Learning Style Inventory for Athletes into Turkish and to examine its construct validity and reliability within a sample of Turkish athletes. Specifically, the study aims to evaluate the construct validity of the inventory using a multitrait–multimethod analytical framework, assess internal consistency reliability, and examine test–retest reliability to determine the temporal stability of learning preference scores. By focusing on the psychometric evaluation of the athlete-specific version, the study aims to address an existing methodological gap in the literature and to provide empirical evidence that can inform future research on learning preferences in sport-related educational settings.

## Materials and methods

### Participants

The sample of the study consisted of athletes from various sports disciplines in Türkiye, of whom 51.9% (*n* = 443) were engaged in individual sports and 48.1% (*n* = 411) in team sports. All participants were actively involved in organized training and competitions across different league levels and had at least 2 years of sport-specific training experience. To account for potential linguistic and cultural variability, participants were recruited using a randomized selection approach across all seven geographical regions of Türkiye. A total of 881 athletes aged between 20 and 29 years (M = 23.63, SD = 1.72) initially participated in the study, of whom 35.4% were female, and 64.6% were male. The regional distribution of participants was as follows: Mediterranean Region (13.2%), Eastern Anatolia (14.1%), Aegean (15.5%), Southeastern Anatolia (13.8%), Central Anatolia (15.0%), Black Sea Region (14.2%), and Marmara Region (14.3%).

The sample included athletes from multiple sports disciplines, including athletics, football, basketball, volleyball, handball, swimming, gymnastics, combat sports, and racket sports. The inclusion of athletes from diverse sports disciplines was intentional and aligned with the study's primary aim: to evaluate the general psychometric properties of the VARK Learning Style Inventory for Athletes rather than to conduct sport-specific comparisons. Accordingly, no minimum sample size per sport discipline was imposed, and athletes from sports with limited representation were retained in the analysis to ensure sample heterogeneity and to support the generalizability of the factor structure across the target athletic population. According to the scoring criteria of the VARK Learning Style Inventory for Athletes, responses from 27 participants who made fewer than 10 selections were excluded from the statistical analyses [35]. Consequently, the final analytical sample consisted of 854 athletes.

### Questionnaire

Two instruments were used for data collection in this study. The first was a personal information form designed in line with the study objectives. This form collected data on participants’ gender, year of birth, geographical region of residence, and type of sport participation (individual or team). The second section utilized the VARK Learning Style Inventory for Athletes, developed by Dunn and Fleming [35].

This version of the VARK model, which seeks to answer the question “How do I learn best?”, consists of 16 items, each offering four response options representing different learning styles: visual, aural, read/write, and kinesthetic (unimodal). These options are embedded in varying combinations across a total of 64 statements, with each statement corresponding to one of the four learning preferences (V, A, R, or K). To ensure the inventory is completed accurately, an important instruction appears above the first item: “Tick the option(s) that best describe your preference. If more than one applies, you may select multiple options.” This instruction emphasizes that individuals may have one or more dominant learning styles and reflects the multi-trait multi-method (MTMM) structure of the inventory. According to the scoring guidelines, responses from participants who marked fewer than 10 items in total out of 64 are excluded from statistical analysis. Scoring is conducted in two stages. Scoring was conducted in two stages. In the first stage, total scores for the Visual (V), Aural (A), Read/Write (R), and Kinesthetic (K) dimensions were calculated and ranked from highest to lowest to identify the dominant learning preference(s). In the second stage, a distance-based “stepping-stone” criterion was applied to classify individuals into four categories unimodal, bimodal, trimodal, or multimodal learning profiles. According to this criterion, if the total sum of scores across the four styles is 10–16, the distance value is 1; 17–22, the distance is 2; 23–30, the distance is 3; and above 30, the distance is 4. The difference between the highest and second-highest scores is compared to the relevant distance value. If the difference is greater, the individual is classified as having a unimodal learning style. If not, the difference between the second and third highest scores is considered; if it exceeds the distance value, the profile is bimodal. If this condition is not met, the third and fourth scores are compared, and if the difference is greater than the distance, the profile is trimodal. If none of these differences exceed the threshold, it is concluded that the individual exhibits a multimodal learning style, using various combinations of seeing, hearing, reading/writing, or doing as part of their learning process [15, 27, 35].

### Procedures

Permission to use the VARK Learning Style Inventory for Athletes was obtained from the copyright holders via email, with approval granted for paper-based administration only. The study was approved by the social and human sciences research ethics Committee of the Bayburt University (approval number: 2020/92) and was conducted according to the Declaration of Helsinki. Written informed consent was obtained from all participants prior to data collection, and participants were informed of their right to withdraw from the study at any time without penalty. In accordance with the criteria established by Chen and Boore, the translation-back translation method was applied [36]. First, the original English version of the inventory was translated into Turkish by four subject matter experts with a high level of proficiency in English. This Turkish version was then back-translated into English by three academic experts in sports sciences and educational measurement. Comparisons were made between the back-translated version and the original inventory to ensure conceptual equivalence and linguistic accuracy. Following this process, a Turkish teacher reviewed the final version for spelling, grammar, and semantic clarity. Minor linguistic corrections were made, and the test-retest reliability of the testlets and items was assessed using a test-retest method with a pilot group of 48 athletes at 4-week intervals. Based on feedback from this pilot group, the final Turkish version of the inventory was completed. The data collection process then commenced. The Demographic Information Form and the VARK Learning Style Inventory for Athletes, along with additional explanatory notes prepared by the researchers, were reproduced and distributed in printed format. Sports clubs were selected to represent different sports disciplines and geographical regions of Türkiye. Clubs with active licensed athletes and regular training programs were contacted to ensure the sample was relevant to the study objectives. During data collection, logistical support was provided by club managers and coaches, who facilitated access to athletes and coordinated the administration of the questionnaires. All data were collected face-to-face using a paper-based method. To ensure procedural standardization across different regions and sports clubs, all coaches and club managers involved in the data collection process were provided with the same written administration guidelines prepared by the researchers. These guidelines specified the standardized instructions to be delivered verbatim to all participants, including explicit emphasis on the option to select more than one response per item. In addition to the written instructions, researchers or trained assistants provided brief, uniform verbal explanations before administration and clarified that multiple options could be selected when applicable. Participants were given the opportunity to ask questions before completing the inventory, and any clarification requests were addressed immediately to ensure an accurate understanding of the response format. This standardized face-to-face administration procedure was applied consistently across all regions to minimize procedural variability and enhance the reliability of the collected data. Before participants completed the forms, verbal explanations were provided regarding the VARK model's application criteria in accordance with the MTMM methodology. After participants completed the forms, the responses were processed, and the data sets were compiled and prepared for statistical analysis.

### Analysis

The VARK Learning Style Inventory for Athletes consists of 16 question blocks, each containing four response options corresponding to the Visual, Aural, Read/Write, and Kinesthetic learning styles. Each response option is scored dichotomously (selected or not selected), resulting in a total of 64 dichotomous items. A testlet is a set of items grouped together as a unit of measurement during test construction, administration, and scoring. In the VARK inventory, each testlet contains items that share a common stem or structural format [25].

In confirmatory factor analysis (CFA) or item response theory (IRT) analyses, it is generally assumed that responses to individual items are statistically independent after accounting for the underlying latent constructs. This assumption, referred to as local item independence, implies that item responses are unrelated after controlling for the latent traits. However, when items are organized into testlets, local dependence may occur, and failure to account for this dependence can result in poor model fit. One proposed solution to address local dependence is to treat each testlet as a single polytomous item and apply a polytomous IRT model. This approach, however, is appropriate only when testlets are unidimensional, meaning they measure a single latent trait. While various IRT models have been developed for unidimensional testlets, multidimensional extensions have also been proposed for more complex measurement structures [25, 37].

The selection of a Multitrait–Multimethod Confirmatory Factor Analysis (MTMM-CFA) framework was theoretically and methodologically driven by the structural characteristics of the VARK Learning Style Inventory for Athletes. Traditional CFA models assume a simple structure and local item independence, which are not fully compatible with instruments composed of interrelated testlets and overlapping content domains. Although alternative approaches such as exploratory structural equation modeling (ESEM) or bifactor models allow for cross-loadings, they do not explicitly model method effects associated with testlet-based item groupings. In contrast, the MTMM-CFA approach enables the simultaneous estimation of trait variance and method-related covariance, thereby providing a more appropriate representation of the inventory’s multidimensional and multimethod nature. The correlated trait–correlated uniqueness (CTCU) specification was therefore preferred, as it allows shared method variance among testlets to be explicitly modeled without inflating trait correlations or compromising construct interpretability. The VARK Learning Style Inventory for Athletes demonstrates a multidimensional and multimethod structure, as each item contains elements associated with more than one learning style. Accordingly, rather than employing traditional exploratory or confirmatory factor analysis models, this study utilized a Multitrait–Multimethod Confirmatory Factor Analysis (MTMM-CFA) approach capable of simultaneously modeling trait variance and method effects associated with testlets. Consistent with the recommendations of Leite et al., the Correlated Trait–Correlated Uniqueness (CTCU) model was adopted, as it has been shown to be particularly suitable for instruments with interrelated dichotomous testlets representing overlapping constructs [25]. The CTCU specification allows correlations among latent traits while accounting for shared method variance at the testlet level, thereby enabling a more accurate estimation of the factorial structure.

Within the MTMM-CFA framework, factor loadings were interpreted as indicators of the strength of association between each testlet and the latent learning style dimensions, rather than as criteria for assigning items or testlets to a single factor, as is common in traditional CFA models. Given the CTCU specification, testlets were permitted to load on multiple latent traits, reflecting the overlapping sensory characteristics inherent in the VARK inventory. Therefore, instances in which a testlet exhibited similar standardized loadings across more than one factor (e.g., comparable loadings on the Visual and Aural dimensions) were not interpreted as problematic cross-loadings or model misspecifications, but rather as theoretically expected outcomes of the multitrait–multimethod structure. In line with recommendations in the MTMM literature, standardized loadings of approximately 0.20 or higher were considered meaningful for interpretation within this modeling approach [25, 37].

**Fig. 1:**
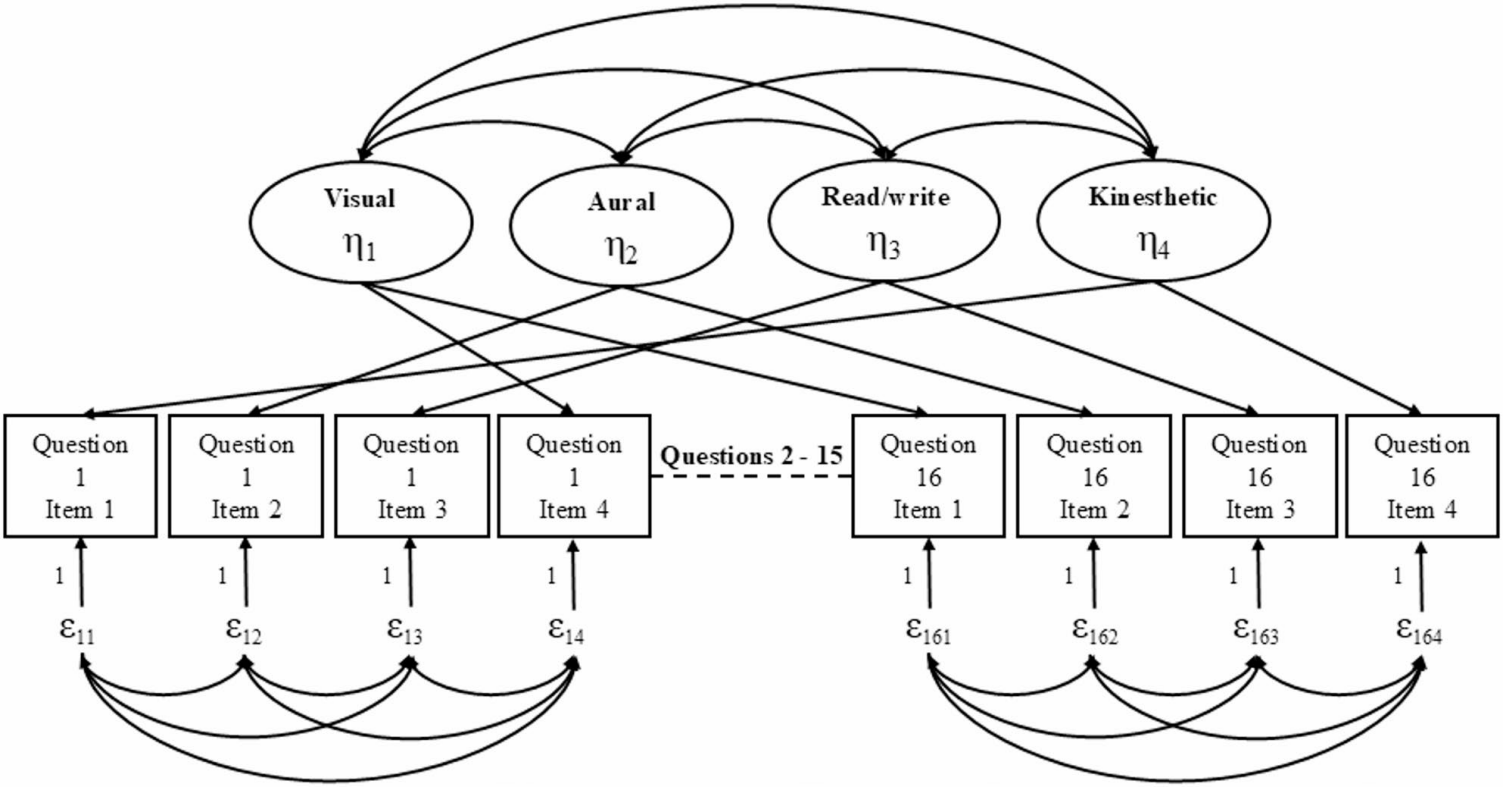
Correlated trait–correlated uniqueness model for VARK (CTCU)

The MTMM-CFA model was used to examine the construct validity of the VARK Learning Style Inventory for Athletes and to estimate factor loadings and inter-factor correlations for the four learning style dimensions, 16 testlets, and 64 dichotomous items. Internal consistency was evaluated using the Kuder–Richardson 20 (KR-20) coefficient, which was preferred over Cronbach’s alpha because the testlets used a dichotomous (0/1) scoring format. The overall model specification and factor structure are presented in Fig. 1. All statistical analyses were conducted using IBM SPSS Statistics (v26) and LISREL (v8.80).

## Results

As part of the psychometric evaluation of the Turkish version of the VARK Learning Style Inventory for Athletes, a pilot study was conducted to assess test–retest reliability over a four-week interval. The findings indicated acceptable to good temporal stability across all four learning style dimensions, suggesting that participants’ learning preferences remained relatively consistent over the four-week interval (*r* = 0.55–0.74; α = 0.71–0.85). The Aural dimension exhibited the highest stability (*r* = 0.742, α = 0.852), while the Kinesthetic dimension showed relatively lower but still acceptable reliability (*r* = 0.551, α = 0.709). These findings provide additional evidence supporting the psychometric robustness and temporal consistency of the Turkish adaptation of the inventory, confirming its suitability for longitudinal applications in sport education research (Table 1).

**Table 1:** Test–retest reliability of the Turkish version of the VARK inventory for athlete

Factor	Test (X̄)	Re-test (X̄)	*r*	α	%95 CI
Visual	6.50 ± 2.44	6.75 ± 2.51	0.694	0.819	0.514 − 0.816
Aural	5.79 ± 2.53	6.10 ± 2.57	0.742	0.852	0.581 − 0.845
Read/Write	5.45 ± 2.27	5.47 ± 2.46	0.661	0.794	0.468 − 0.796
Kinesthetic	8.62 ± 1.96	8.93 ± 1.82	0.551	0.709	0.317 − 0.717

*n* = 48 (pilot study), α: Reliability coefficient, r: Correlation, 95% CI = %95 Confidence Interval

**Fig. 2:**
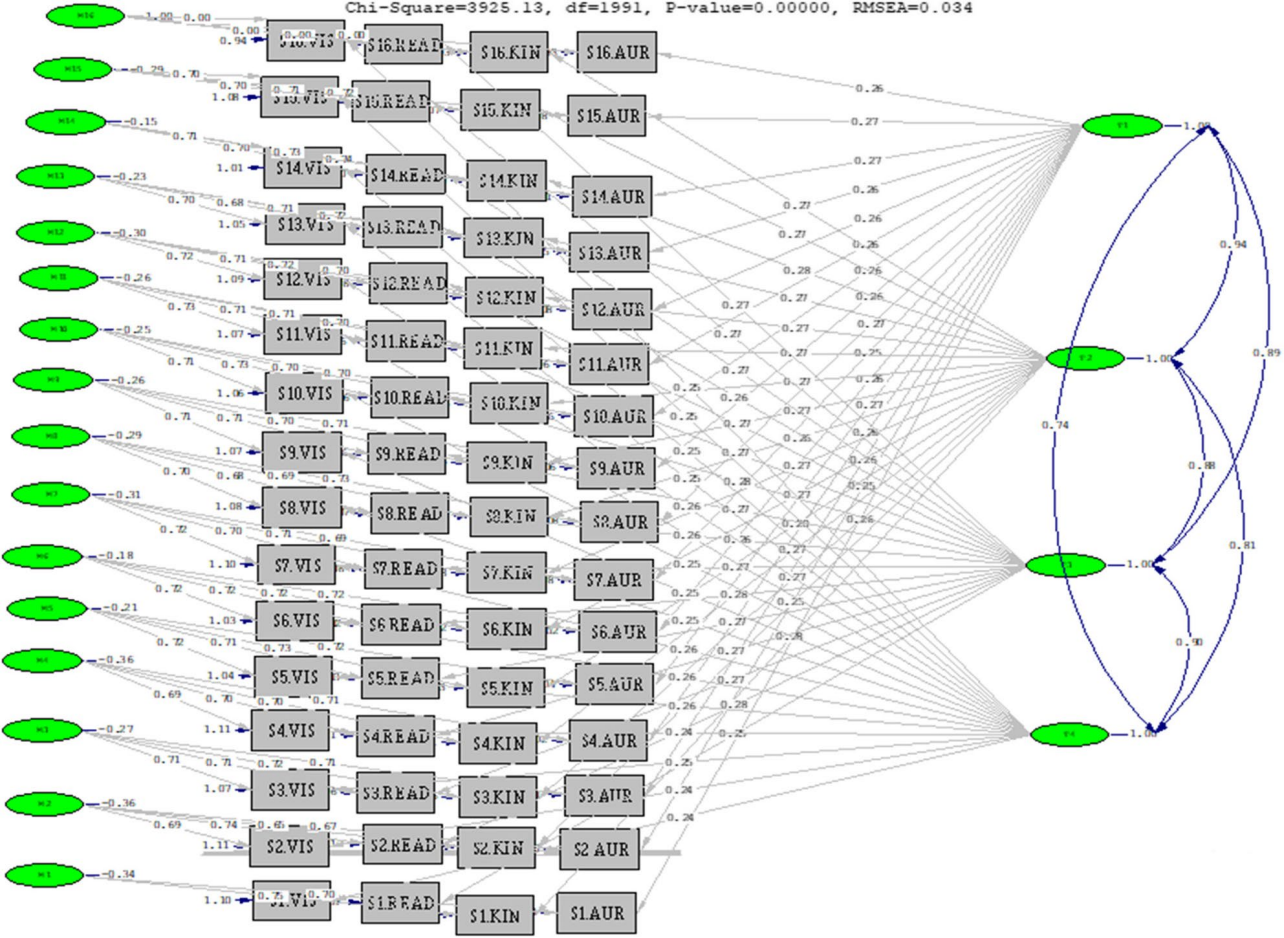
Path diagram of the final CTCU Model for the Turkish VARK inventory

Based on the fit index criteria recommended by Kline [38], the analysis of the Turkish VARK Learning Style Inventory for Athletes using the CTCU model demonstrated excellent psychometric properties, with χ²/df (3925.13/1991) = 1.97, which falls within the excellent range. The additional fit indices further confirmed the structural validity of the measurement model, with RMSEA = 0.034 indicating excellent fit and the remaining indices all meeting acceptable thresholds (GFI = 0.91, AGFI = 0.87, CFI = 0.90, NNFI = 0.90, IFI = 0.90, SRMR = 0.05, PNFI = 0.74, PGFI = 0.84). These comprehensive results substantiate the construct validity of the four-factor VARK structure in its Turkish adaptation and confirm the inventory’s adequate psychometric qualities. The complete set of model fit indices is systematically presented in Table 2, while the structural relationships are visualized in the path diagram shown in Fig. 2.

**Table 2:** Model fit indices for the Turkish version of the VARK inventory for athlete

Fit indices	The perfect fit criteria	The acceptable fit criteria	Four factor model	Result
χ^2^/sd	0 ≤ χ^2^/sd ≤ 2	2 ≤ χ^2^/sd ≤ 3	3925.13/1991 = 1.97	Perfect
GFI	0.95 ≤ GFI ≤ 1.00	0.90 ≤ GFI ≤ 95	0.91	Acceptable
AGFI	0.90 ≤ AGFI ≤ 1.00	0.85 ≤ AGFI ≤ 0.90	0.87	Acceptable
CFI	0.95 ≤ CFI ≤ 1.00	0.90 ≤ CFI ≤ 0.95	0.90	Acceptable
NNFI	0.95 ≤ NNFI ≤ 1.00	0.90 ≤ NNFI ≤ 0.95	0.90	Acceptable
IFI	0.95 ≤ IFI ≤ 1.00	0.90 ≤ IFI ≤ 0.95	0.90	Acceptable
RMSEA	0.00 ≤ RMSEA ≤ 0.05	0.05 ≤ RMSEA ≤ 0.08	0.04	Perfect
SRMR	0.00 ≤ SRMR ≤ 0.05	0.05 ≤ SRMR ≤ 0.10	0.05	Acceptable
PNFI	0.95 ≤ PNFI ≤ 1.00	0.50 ≤ PNFI ≤ 0.95	0.74	Acceptable
PNGI	0.95 ≤ PGFI ≤ 1.00	0.50 ≤ PGFI ≤ 0.95	0.84	Acceptable

*GFI* Goodness of fit index, *AGFI* Adjusted goodness of fit index, *CFI* Comparative fit index, *NNFI* Non-normed fit index, *IFI* Incremental fit index, *RMSEA* Root mean square error of approximation, *SRMR* standardized root mean square residual, *PNFI* Parsimony normed fit index, *PNGI* parsimony goodness of fit index, *n* = 854

According to the CTCU model, the standardized factor loadings of the items in the Turkish adaptation of the VARK Learning Style Inventory for Athletes ranged from 0.236 to 0.258 for the Visual factor, 0.245 to 0.271 for Aural, 0.253 to 0.282 for Read/Write, and 0.248 to 0.284 for the Kinesthetic factor. This pattern of loadings provides key evidence for the instrument’s factorial validity. In addition, an examination of the inter-factor correlations revealed strong to very strong relationships among the learning style factors, further supporting the internal construct validity of the model. Specifically, the correlation between Visual and Aural styles was 0.736 (strong), while Kinesthetic–Visual was 0.814, Aural–Read/Write was 0.893, Visual–Read/Write and Read/Write–Kinesthetic were 0.898, and the highest correlation was observed between Aural and Kinesthetic, at 0.943, indicating a very strong association (Table 3).

**Table 3:** Factor loadings and inter-factor correlations from the CTCU model

	Visual	Aural	Read/Write	Kinesthetic
Q1	0.236	0.259	0.253	0.284
Q2	0.244	0.245	0.282	0.248
Q3	0.251	0.261	0.269	0.274
Q4	0.244	0.261	0.267	0.265
Q5	0.255	0.266	0.270	0.278
Q6	0.255	0.265	0.276	0.273
Q7	0.256	0.253	0.266	0.270
Q8	0.247	0.268	0.261	0.262
Q9	0.252	0.263	0.273	0.266
Q10	0.252	0.256	0.278	0.266
Q11	0.258	0.258	0.272	0.269
Q12	0.255	0.257	0.272	0.272
Q13	0.249	0.265	0.260	0.270
Q14	0.252	0.271	0.268	0.278
Q15	0.246	0.266	0.268	0.268
Q16	0.248	0.261	0.267	0.266
Aural	0.736	-		
Read/Write	0.898	0.893	-	
Kinesthetic	0.814	0.943	0.898	-

An examination of the reliability properties for the Turkish version of the VARK Learning Style Inventory for Athletes (Table 4) revealed KR-20 coefficients ranging from 0.574 to 0.623 across all learning styles. These results indicate acceptable internal consistency for the inventory as a psychometric instrument. Among the four learning style dimensions, the highest reliability coefficient was found for both the Aural and Read/Write styles (0.623), while the lowest was observed for the Kinesthetic style (0.574). On average, KR-20 values approximated 0.60, confirming the measurement reliability of the Turkish adaptation and supporting its consistency in assessing athletes’ learning preferences.

**Table 4:** Factor loadings and inter-factor correlations from the CTCU model

Factors	Item number	KR-20
Visual	16	0.602
Aural	16	0.623
Read/Write	16	0.623
Kinesthetic	16	0.574

## Discussion

The VARK Learning Style Inventory for Athletes is a sensory modality-based tool designed to identify individual learning preferences. In sports education, aligning instructional methods with these preferences is often hypothesized to enhance motor skill development, strategic thinking, and athletic performance. However, empirical evidence supporting the direct impact of learning styles on performance outcomes remains limited is debated in the scientific literature [20, 21]. Furthermore, beyond its applied relevance for sport pedagogy, this study provides an important methodological contribution by demonstrating the utility of the Multitrait–Multimethod Confirmatory Factor Analysis approach in validating multidimensional learning inventories. The use of the correlated trait–correlated uniqueness (CTCU) model offers a more rigorous framework for distinguishing trait variance from method effects, thereby improving measurement precision and construct validity in sport-related psychometric assessments [12, 13]. From a methodological standpoint, the application of MTMM-CFA models has been increasingly recommended in sport and exercise sciences when instruments are characterized by overlapping constructs and shared method variance [39].

The temporal stability findings obtained from the pilot study indicate that the Turkish version of the VARK Learning Style Inventory for Athletes demonstrates an acceptable level of test–retest reliability, with stability coefficients comparable to those reported in previous psychometric adaptations of perceptual learning and learning preference instruments (Table 1). Similar levels of moderate temporal stability have been reported in studies examining learning style and sensory preference measures, suggesting that such constructs reflect relatively stable tendencies while remaining open to experiential influences [23, 40]. In sport and physical activity contexts, learning preferences are shaped not only by cognitive characteristics but also by accumulated training experiences and coaching practices. In this regard, the relatively higher temporal stability observed in the auditory dimension is consistent with prior findings indicating that verbal instruction and auditory feedback constitute persistent and frequently utilized channels in coaching environments [23, 41]. Conversely, the comparatively lower yet still acceptable stability of the kinesthetic dimension aligns with the notion that body-based learning preferences are more sensitive to contextual demands, task constraints, and variations in training load, as also noted in applied sport learning research [40]. Taken together, these findings support theoretical perspectives suggesting that learning preferences should not be conceptualized as rigid traits, but rather as moderately stable patterns that may evolve over time in response to environmental and experiential factors. This interpretation is consistent with contemporary views in motor learning research, which emphasize adaptability and context sensitivity as defining characteristics of learning-related individual differences in sport [16]. Within this framework, the observed temporal stability provides empirical support for the use of the Turkish VARK Athletes Inventory in longitudinal research designs, where monitoring changes in learning preferences may offer valuable insights for adaptive and individualized coaching and instructional practices in sport settings.

The analysis of the model fit indices, conducted via the CTCU model within the MTMM-CFA framework, provided strong evidence for the construct validity of the Turkish version of the VARK Learning Style Inventory for Athletes (Table 2). The psychometric properties observed in this study are consistent with those reported in previous cross-cultural and population- specific adaptations of the VARK model. For instance, Düzgün’s study with teachers, which utilized the general version of the inventory, also confirmed the four-factor structure and reported comparable fit indices [33]. Similarly, Leite, Svinicki, and Shi validated the four-factor structure and established sound psychometric properties for the original English version [25]. This consistency across different sample groups, including teachers, general students, and now Turkish athletes, and across languages, reinforces the robustness and cross-cultural stability of the VARK model’s construct validity. Cross-validation across distinct populations is widely regarded as a critical criterion for establishing the generalizability of psychometric instruments in applied research contexts [38]. Therefore, the current findings substantiate that the athlete-specific version also possesses strong psychometric qualities, supporting its use for research and applied purposes with Turkish athletes.

The evaluation of the model fit indices for the Turkish adaptation of the VARK Learning Style Inventory for Athletes, based on the criteria recommended by Kline [38], indicates that the proposed MTMM-CFA model provides an adequate and theoretically coherent representation of the data. Rather than relying on individual fit statistics in isolation, the overall pattern of absolute, incremental, and parsimony-adjusted indices suggests that the CTCU specification achieved a balanced fit while appropriately accounting for both trait variance and method effects inherent in the VARK structure. From a psychometric perspective, this finding is particularly relevant given the multidimensional and overlapping sensory characteristics of the inventory. Previous studies applying the VARK framework in athletic and applied learning contexts have similarly emphasized the importance of evaluating model fit holistically when multimethod effects are present [7, 8]. In this respect, the observed fit pattern supports the construct validity of the four-factor VARK structure within the Turkish athletic context and aligns with evidence reported in earlier validation studies conducted in different cultural settings. Taken together, these results indicate that the satisfactory model fit was achieved without unnecessary model complexity, as also reflected in acceptable parsimony indices. Consequently, the Turkish version of the VARK Learning Style Inventory for Athletes can be considered to exhibit sound psychometric qualities for use in both research and applied sport settings. Moreover, the consistency of these findings with prior studies further supports the cross-cultural stability of the underlying factor structure and reinforces the potential utility of the inventory for assessing learning preferences in diverse athletic populations [7, 8].

Analysis of the factor structure provided further evidence for the psychometric adequacy of the Turkish adaptation. The standardized item factor loadings ranged from 0.236 to 0.258 for the Visual factor, 0.245 to 0.271 for Aural, 0.253 to 0.282 for Read/Write, and 0.248 to 0.284 for Kinesthetic (Table 3). All loadings exceeded the 0.20 threshold, which is considered acceptable for measurement tools of this nature [42] and confirms that each item meaningfully represents its intended latent construct. This pattern of loadings substantiates the structural validity of the inventory and provides critical evidence for its construct validity in the Turkish athletic context [43]. Within MTMM-based validation studies, such loading magnitudes are considered theoretically meaningful when method effects are explicitly modeled, as in the present study (39). From a measurement perspective, the present findings provide empirical support for the construct validity and factorial stability of the VARK Athlete model within a culturally distinct sample. The replication of the four-factor structure under a correlated trait correlated uniqueness specification suggests that learning preferences operate as related but distinguishable latent traits. This evidence strengthens the generalizability of the VARK’s multidimensional measurement model and underscores its suitability for sport-specific educational assessment. Nevertheless, factor loadings of this magnitude should be interpreted with caution, as relatively small standardized coefficients may limit the precision of individual-level score interpretations and reduce sensitivity for fine-grained diagnostic purposes. In this respect, the present findings primarily support the inventory’s suitability for group-level research and psychometric evaluation rather than for high-stakes individual assessment or decision-making.

An examination of the inter-factor correlations provided further validation of the inventory’s psychometric properties. The findings revealed a particularly strong relationship between the Aural and Kinesthetic learning styles (*r* = 0.943), a pattern consistent with previous psychometric evaluations of the VARK model that reported similarly high correlations between sensory modalities [25]. Furthermore, the strong Visual–Aural correlation (*r* = 0.736) aligns with established literature on integrated sensory processing in learning [32–34], while the robust Visual–Kinesthetic association (*r* = 0.814) reflects athletes’ documented dependence on visual feedback during motor learning processes [18]. High inter-factor correlations in athletic populations may reflect functional integration of sensory systems rather than construct redundancy, particularly in skill acquisition contexts requiring coordinated perceptual motor processing [17]. This network of strong correlations powerfully demonstrates the integrated nature of sensorimotor processing in athletic contexts, where observation and physical practice are intrinsically intertwined. Although the correlations among factors were high, these results are conceptually consistent with the multimodal nature of the VARK framework. The strong inter-factor associations reflect the integrated sensory processing required in athletic learning contexts, rather than statistical redundancy or construct overlap. This finding supports the interpretation that the inventory captures a coherent but multidimensional structure of perceptual learning preferences among athletes. Similar psychometric adaptation studies conducted in the United States of America, Türkiye, and New Zealand also reported moderate-to-high inter-factor correlations and satisfactory model fit indices, indicating cross-cultural robustness of the VARK framework. However, the present study extends this evidence by confirming these properties in a specifically athletic population, where sensory–motor integration is inherently stronger. This context-specific validation enriches the existing literature by demonstrating that learning style constructs retain factorial coherence even in populations characterized by high perceptual interactivity [15, 25, 33]. From a practical perspective, these psychometric findings carry significant implications for coaching pedagogy. The demonstrated multimodality suggests that the VARK inventory’s primary value lies not in rigidly categorizing athletes but in promoting pedagogical awareness and instructional variety among coaches, encouraging methods that strategically blend demonstration, physical practice, and verbal feedback [44, 45]. This approach is supported by meta-analysis evidence indicating that coach education programs focusing on tailored pedagogical strategies yield moderate-to-large effects on coaching effectiveness [45]. Therefore, when used as a reflective tool in coach development, the inventory can guide the design of training sessions that create more inclusive and effective learning environments by aligning with athletes’ natural multimodal learning processes. Importantly, the practical use of the VARK inventory should not be interpreted as advocating rigid learning-style matching or deterministic instructional prescriptions. Consistent with critical perspectives in the literature, the inventory is best conceptualized as a reflective and descriptive tool that may enhance pedagogical awareness rather than as a prescriptive framework for optimizing performance outcomes.

An examination of the reliability properties of the Turkish adaptation, in line with Lujan and DiCarlo’s emphasis on cross-cultural linguistic considerations [34], indicated that the KR-20 coefficients for the subscales ranged from 0.574 to 0.623, yielding an overall average of approximately 0.60 (Table 4). These values confirm that the internal consistency of the adapted instrument is within an acceptable range, thereby supporting one of its fundamental psychometric properties. Reliability coefficients of this magnitude are commonly reported in multidimensional learning preference instruments, particularly when constructs reflect heterogeneous experiential domains [46, 47]. Specifically, the Aural and Read/Write subscales demonstrated the highest reliability coefficients (0.623), whereas the Kinesthetic subscale yielded the lowest (0.574), suggesting a relatively comparable, though varied, level of consistency across the inventory’s dimensions. While these KR-20 coefficients are deemed acceptable, their moderately moderate magnitude invites further methodological reflection. The relatively lower reliability coefficient observed for the kinesthetic subscale (KR-20 = 0.574) warrants a sport-specific interpretation. While often categorized under a single label, ‘kinesthetic learning’ likely manifests in fundamentally different ways across diverse sports disciplines. For an athlete in an aesthetic sport like gymnastics or diving, kinesthetic perception is synonymous with fine-tuned proprioception and air awareness. In contrast, for an athlete in football or basketball, it relates to dynamic balance, evading opponents, and executing skills under pressure. For a combat sport athlete, it involves sensing an opponent’s force and weight distribution. The VARK items, which ask about general learning preferences (e.g., ‘I prefer to learn by doing’), may be too broad to capture these nuanced, discipline-specific expressions of kinesthetic processing. Consequently, athletes from different sports may interpret the same kinesthetic item through the lens of their unique motor demands, introducing variability that attenuates the reliability score. This finding suggests that future research should explore whether sport-specific modifications to the kinesthetic items could enhance the scale’s precision for particular athletic populations.

A comparison of the reliability findings with the existing literature reveals that the KR-20 and Cronbach’s Alpha coefficients for various versions of the VARK Learning Style Inventory typically fall within the range of 0.60 to 0.75 [15, 25, 33]. The KR-20 coefficients obtained in the present study align substantially with this established psychometric profile. However, the relatively lower reliability coefficient observed for the kinesthetic learning style in this athlete-specific adaptation suggests potential measurement variability unique to this dimension. The kinesthetic style, characterized by its reliance on movement and direct experience, inherently encompasses a more subjective learning process [24]. Consequently, the discrepancy between the higher kinesthetic reliability coefficients reported in general VARK versions and the lower coefficient identified in this study may be attributable to distinctive characteristics of athletic populations, including diverse sport-specific profiles, the heterogeneity of sports disciplines sampled, and the central role of embodied, experiential learning in athletic training contexts. Based on these psychometric observations, it is recommended that future investigations specifically examine the kinesthetic learning style across different sport disciplines to elucidate the factors underlying its differential reliability performance.

### Limitations

Several limitations of the present study should be acknowledged. First, although the sample included athletes from a wide range of sports, the analyses were conducted at an aggregate level rather than stratified by specific sports. As a result, potential differences in learning preferences associated with sport-specific demands, training environments, or motor task characteristics could not be examined. In addition, the relatively homogeneous age range of the sample (20–29 years) limits the generalizability of the findings to younger or older athletic populations, where learning preferences may be shaped by different developmental, cognitive, and experiential factors.

Second, the heterogeneous composition of the sample, while appropriate for the primary aim of psychometric validation, limits the extent to which the findings can be generalized to individual sport disciplines. Future studies may benefit from examining the psychometric properties of the VARK Learning Style Inventory for Athletes within more homogeneous sport-specific samples to explore potential variations in factor structure or learning preference profiles.

Finally, although temporal stability was examined, longitudinal research designs with extended follow-up periods may provide further insight into how learning preferences evolve over time in response to training intensity, competitive level, and coaching practices. In addition, the moderate level of internal consistency observed in some subscales, particularly the kinesthetic dimension, should be considered a limitation when interpreting the stability and precision of learning preference scores. Although such coefficients are common in multidimensional preference-based instruments, they suggest that the scale may be more appropriate for exploratory and research-oriented applications than for individual-level profiling.

## Conclusions

This study examined the psychometric properties of the Turkish version of the VARK Learning Style Inventory for Athletes and demonstrated that the inventory is a psychometrically sound instrument for identifying the learning styles of Turkish athletes. The results of the Multitrait–Multimethod Confirmatory Factor Analysis (MTMM-CFA) confirmed that the four-factor structure of the inventory remains valid in its Turkish adaptation, with model fit indices falling within excellent and acceptable ranges. In particular, fit indices such as χ2/df, RMSEA, GFI, AGFI, and CFI provided strong evidence for the construct validity of the inventory. Moreover, the standardized factor loadings indicated that the four learning styles in the VARK model are significantly interrelated and are consistently integrated within learning processes.

The reliability analyses also demonstrated satisfactory psychometric characteristics, with KR-20 reliability coefficients falling within acceptable levels. However, the kinesthetic learning style exhibited lower reliability coefficients than the other VARK dimensions. This finding may be attributed to the subjective nature of the kinesthetic style and the diversity of athletic disciplines represented in the sample. It suggests that kinesthetic learning may be a more variable modality, with greater individual differences in measurement likely to emerge based on athletes’ varied experiences and contexts.

Comparative analysis with existing literature indicates that both general versions of VARK and this Turkish adaptation developed specifically for athletes serve as psychometrically valid tools for identifying learning styles across different cultures and sample groups. These results support the cross-cultural robustness of the VARK model and suggest that it can be effectively used as a measurement instrument in assessing learning styles within athletes’ learning processes.

It is also recognized that learning styles are dynamic constructs that may evolve with experience. Therefore, while the psychometric soundness of an inventory is a prerequisite for its meaningful application, the results it provides should be interpreted with caution. The findings from the VARK inventory can offer valuable insights for coaches seeking to diversify their instructional approaches, but they should not be considered the sole determinant of training design. Effective learning in athletes is multifactorial, dependent not only on learning preferences but also on well-designed training methods, appropriate practice content, athlete motivation, and supportive environments. Consequently, tools like the VARK should be integrated as one component within a broader, evidence-based pedagogical framework, rather than used in isolation to dictate instruction. Overall, the study advances measurement methodology in sport and exercise psychology by demonstrating how multimethod factor-analytic models can enhance the psychometric rigor of learning style assessments.

### Practical implications

The adaptation of the VARK Learning Style Inventory for Athletes into Turkish provides coaches, educators, and sports scientists with a psychometrically validated assessment tool for developing individualized instructional strategies that account for personal differences. By identifying athletes’ dominant or multimodal learning styles, the inventory helps coaches design tailored training. This makes it easier to adapt drills, tactical sessions, and psychological preparation to each athlete’s needs. For instance, athletes with dominant visual preferences may benefit more from video-based performance feedback, while those with strong kinesthetic styles may improve faster through simulation drills and on-field experiential learning. Coaches can therefore adapt the balance of instructional methods depending on the athlete’s profile, which can improve learning retention and accelerate skill transfer to competition settings. Accordingly, coaches and educators can structure visual, auditory, kinesthetic, or read/write-based content in alignment with athletes’ perceptual preferences, thereby enhancing learning retention and performance improvement. For example, in team sports such as football or basketball, visual learners may benefit from tactical board sessions and video analysis, whereas kinesthetic learners may gain more from repetitive situational drills that simulate match conditions. In individual sports such as athletics or swimming, auditory learners can respond well to rhythm-based feedback and verbal cueing, while read/write-oriented athletes may prefer structured training logs and reflective journals. These practical applications show how coaches can flexibly integrate the inventory into daily training routines. Furthermore, the inventory’s demonstrated psychometric properties support the broader application of the VARK model in sport-specific learning contexts and contribute to the establishment of evidence based pedagogical practices. Considering individual learning differences when planning training holds the potential to enhance athletic performance and increase athlete engagement. In practical terms, this offers coaches a methodologically grounded approach to adapt training sessions, thereby facilitating more efficient skill acquisition and longer-term retention.

## Data Availability

The data used to support the findings of this study are available from the corresponding author upon request.
